# Is Monkeypox Being Underdiagnosed in Countries with More Stigmatizing Attitudes towards Men Who Have Sex with Men? A Simple Ecological Analysis

**DOI:** 10.3390/epidemiologia3030028

**Published:** 2022-08-18

**Authors:** Chris Kenyon

**Affiliations:** 1 Department of Clinical Sciences, Institute of Tropical Medicine, 2000 Antwerp, Belgium; ckenyon@itg.be; 2 Department of Medicine, University of Cape Town, Cape Town 7701, South Africa

**Keywords:** monkeypox, MSM, screening, structural stigma, chlamydia, syphilis

## Abstract

It is not known why the recent outbreak of monkeypox (MPX) has been more extensive in certain European countries than others. Previous studies have found that European countries with more stigmatizing attitudes to homosexuality have more undiagnosed HIV infections in men who have sex with men (MSM). We hypothesized that MPX in MSM may be underdiagnosed in European countries with more stigmatizing attitudes to homosexuality and less access to sexually transmitted infection (STI) testing for MSM. To test this hypothesis, we used Spearman’s correlation to assess if the national incidence of MPX in European countries was negatively associated with the intensity of screening for STIs and a composite indicator of Lesbian Gay Bisexual Transgender Intersex (LGBTI) rights (the Rainbow Index). We found that the national cumulative incidence of MPX was positively correlated with the intensity of chlamydia/gonorrhoea screening (rho 0.68, *p*-value < 0.0001), syphilis screening (rho 0.62, *p*-value < 0.0001), and the Rainbow Index (rho 0.65, *p*-value < 0.0001). Our analysis thus suggests caution is required in interpreting the relatively lower incidence of MPX reported from several Eastern European countries. A key limitation of this analysis is that the incidence of MPX was calculated in the whole population and not limited to the MSM population.

## 1. Introduction

**Figure 1:**
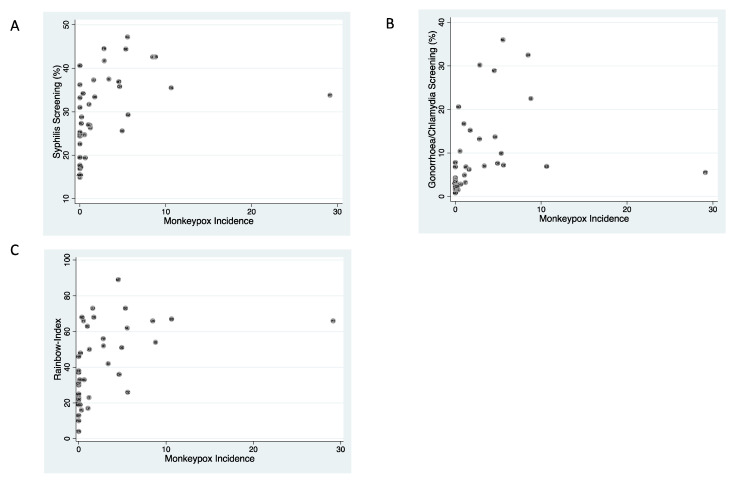
Scatter plot of country-level cumulative incidence of MPX (cases per million 6 May 2022–21 June 2022) and intensity of syphilis screening (**A**), chlamydia/gonorrhoea screening (**B**) and the Rainbow Index (**C**).

Monkeypox (MPX) is a viral zoonosis responsible for outbreaks typically limited to Central and West Africa [1]. Since 6 May 2022, over 36,500 cases of monkeypox (MPX) have been confirmed from countries around the world (https://ourworldindata.org/monkeypox accessed on 16 August 2022). This outbreak is novel not only in its geographic spread but also because for the first time it appears to be predominantly spread amongst men who have sex with men [1,2]. In the United Kingdom, for example, 151 out of 152 MPX cases with available data reported being MSM, and one individual declined to answer the question [3]. Although MPX was first noted in Europe and has spread to countries in all regions of Europe, this spread has not been uniform, with Portugal, the United Kingdom, and Spain reporting particularly high incidences and countries in eastern Europe having lower incidence (Figure 1) [1,2,3,4]. The first reports of MPX were from Portugal and the United Kingdom [1,4]. This distribution may thus be the result of the MPX virus having been introduced in these high incidence countries. Alternatively, the spread of the MPX virus may have been missed in European countries with lower reported incidence. The less-virulent West African MPX clade is responsible for the current outbreak [1,2,3]. This clade is less virulent and frequently presents with localized anogenital or oral lesions with minimal systemic symptoms [1,5]. Such presentations may be misdiagnosed as other more common causes of oro-genital ulceration, such as herpes virus infections [1,2,4]. The proportion of MPX infections correctly diagnosed may vary per country. In part this may be related to national variations in the opportunities for MSM to be tested for STIs in a non-discriminatory environment [6]. For example, the percent of men reporting being screened for various STIs annually varies widely between countries in Europe [6]. The same is true for access to HIV pre- and post-exposure prophylaxis and stigmatizing attitudes towards homosexuality [6,7]. Studies have found that European countries with more stigmatizing attitudes to homosexuality have poorer STI prevention provision and higher risk behaviour in MSM [7,8]. Two studies have found a positive association between national levels of stigma and undiagnosed HIV-infection in MSM in European countries [8,9]. These insights led us to hypothesize that MPX in MSM may be underdiagnosed in European countries with more stigmatizing attitudes to homosexuality and less access to STI testing for MSM. The aim of this study was to assess if the national incidence of MPX was negatively associated with the intensity of screening for STIs and a composite indicator of LGBTI rights.

AMK = Albania/Montenegro/Kosovo, AT = Austria, BY = Belarus, BE = Belgium, BA = Bosnia & Herzegovina, BG = Bulgaria, HR = Croatia, CY = Cyprus, CZ = Czech Republic, DK = Denmark, EE = Estonia, FI = Finland, FR = France, DE = Germany, GR = Greece, HU = Hungary, IS = Iceland, IE = Ireland, IT = Italy, LV = Latvia, LT = Lithuania, LU = Luxembourg, MT = Malta, MD = Moldova, NL = Netherlands, MK = North Macedonia, NO = Norway, PL = Poland, PT = Portugal, RO = Romania, RU = Russia, RS = Serbia, SK = Slovakia, SI = Slovenia, ES = Spain, SE = Sweden, CH = Switzerland, TR = Turkey, UA = Ukraine, UK = United Kingdom.

## 2. Methods

### 2.1. Data Sources

MPX: The national cumulative incidence (6 May 2022 to 21 June 2022) of MPX per million was calculated using MPX incident data from https://ourworldindata.org (accessed on 22 June 2022) and population size data from the United Nations population estimates for 2020 https://population.un.org/wpp/Download/Standard/Population/ (accessed on 22 June 2022).

#### STI Screening

Country-level screening data were obtained from 40 countries participating in the European Men-Who-Have-Sex-With-Men Internet Survey (EMIS) 2017 (Table 1) [6]. The 2017 EMIS was an internet-based survey that recruited 127,000 European MSM and used a self-interviewing format to characterize variables such as sexual behavior and STI screening. The study methodology has been described elsewhere [6] and we obtained our data from one of these study reports [10].

Intensity of chlamydia/gonorrhoea screening: Being screened for chlamydia/gonorrhoea was defined as reporting a test on a urine/urethral swab or an anal swab as part of STI testing in the previous 12 months as well as not reporting any symptoms compatible with chlamydia/gonorrhoea [10].

Intensity of syphilis screening: Having been screened for syphilis, was defined as the respondent reporting no symptoms at the time of their most recent test as well as reporting a blood-based test as part of STI testing in the previous twelve months [10].

For both these variables, the national intensity of screening per country was defined as the percent of respondents per country that reported this type of screening in the prior 12 months. The reason for choosing chlamydia/gonorrhoea and syphilis as measures of intensity of STI screening was related to data availability. The EMIS provides screening intensity data for these three bacterial STIs. EMIS also asks about testing for HIV and viral hepatitis. We did not use this data because the screening intensity of these infections is affected by the proportion of the population who are vaccinated (hepatitis A and B) or chronically infected (HIV, hepatitis B, C).

Rainbow Index 2020: The Rainbow Index is compiled by the International Lesbian, Gay, Bisexual, Transgender, and Intersex Association of Europe as way to rank countries based on the rights and protections provided to lesbian, gay, bisexual, transgender, and intersex (LGBTI) persons [11]. It is a composite indicator that includes legal protection, hate speech, right to marriage and access to appropriate health care. We used data from the 2020 report. The indicator ranges from 0 (few rights) to 100 (well-protected rights) and was previously shown to be negatively associated with undiagnosed HIV infections in European countries [9].

### 2.2. Data Analysis

We used Spearman’s correlation coefficient to assess the association between national MPX incidence, the intensity of each type of STI screening and the Rainbow Index. The statistical analysis was performed using Stata/MP v16.

## 3. Results

Data was available for 40 European countries. The cumulative incidence of MPX varied from 0 to 29 cases/million (median 1.0, interquartile range (IQR) 0–4.6; Figure 1; Table 1). There were marked variations in the Rainbow Index (range 4–89, median 38, IQR 23–63). Variations in national screening intensity for chlamydia/gonorrhoea (range 0.8–36%, median 5.8%, IQR 2.5–11.8%) and syphilis (range 14.9–47.2%, median 30.2%, IQR 24.7–36.6%) were also evident.

### Correlation between MPX, Screening Intensity, and Rainbow Index

The cumulative incidence of MPX was positively correlated with the intensity of chlamydia/gonorrhoea screening (rho 0.68, *p*-value < 0.0001), syphilis screening (rho 0.62, *p*-value < 0.0001), and the Rainbow Index (rho 0.65, *p*-value < 0.0001; Figure 1). The Rainbow Index was, in turn, positively correlated with both intensity of chlamydia/gonorrhoea screening and syphilis screening (rho 0.67, *p*-value < 0.0001; rho 0.44, *p*-value = 0.005, respectively).

## 4. Discussion

In our analysis, countries with more stigmatizing attitudes to homosexuality had lower reported rates of screening for STIs and a lower incidence of MPX. There are a number of possible explanations for these findings. As noted above, MPX may have entered MSM sexual networks in Western Europe and it may simply be a question of time before reported incidence increases in Eastern European countries. Alternatively, differences in sexual behaviour, especially at large festival-type events that may have played a role in generating the current outbreaks, may mean that MPX is able to spread more extensively in certain countries than others [2,4]. There may, however, be important lessons to learn from other STIs. We now have good evidence that the spread of HIV, and other STIs, has not been less extensive in countries with more stigmatizing attitudes to homosexuality. What does differ is that HIV is more likely to spread undetected [8,12]. A number of studies have thus confirmed a strong link between stigma and STI and HIV risk at both individual and population level [12,13,14,15,16,17].

There are a number of study limitations in addition to those mentioned above. It is a purely ecological analysis and thus susceptible to the ecological inference fallacy. We also calculated incidence in the whole population and not limited to the MSM population.

A number of pathways have been proposed to explain the link between stigma and STI/HIV risk. Homophobia and stigma in a society and its health providers could discourage MSM from presenting for testing, and if they do so may reduce the probability of them receiving optimal diagnostic testing and care [7,8,13,16,17]. MSM may also be less likely to report their sexual orientation to providers, impairing quality of care and surveillance of STIs [13,17]. Our analysis thus suggests caution is required in interpreting the relatively lower incidence of MPX reported from several Eastern European countries. Furthermore, it adds evidence to previous calls to support efforts to reduce stigmatization of homosexuality and improve access to STI services in all countries, but particularly so in countries with low scores on the Rainbow Index [16,17].

## Figures and Tables

**Table 1:** Country-level cumulative incidence of monkeypox (MPX, cases per million 6 May 2022–22 May 2022) and intensity of STI screening and value of the Rainbow Index.

Country	Chlamydia/Gonorrhoea Screening (%)	MPX Incidence	Syphilis Screening (%)	Rainbow Index
Albania/Montenegro/Kosovo	3.1	0	15.5	NA
Austria	6.8	1.2	26.3	50
Belarus	7.8	0	33.2	13
Belgium	9.9	5.3	44.4	73
Bosnia & Herzegovina	2.6	0	17.7	37
Bulgaria	2.2	0	24.9	20
Croatia	4.3	0	24.4	46
Cyprus	3.4	0	36.2	31
Czechia	7.2	5.6	29.3	26
Denmark	15.2	1.7	33.4	68
Estonia	3	0	22.6	38
Finland	10.4	0.5	24.7	66
France	13.2	2.8	44.5	56
Germany	7.6	4.9	25.6	51
Greece	2.3	0.2	28.8	48
Hungary	2.8	0.6	19.4	33
Iceland	22.5	8.8	42.6	54
Ireland	30.2	2.8	41.7	52
Italy	3.2	1.2	26.9	23
Latvia	4.9	1.1	31.7	17
Lithuania	1.9	0	14.9	23
Luxembourg	6.2	1.6	37.3	73
Malta	28.9	4.5	36.9	89
Moldova	0.8	0	40.6	19
Netherlands	36	5.5	47.2	62
North Macedonia	2.3	0	19.5	25
Norway	20.6	0.4	34.2	68
Poland	1.5	0.3	24.7	16
Portugal	5.5	29.1	33.8	66
Romania	2	0.2	27.3	19
Russia	6.8	0	33.2	10
Serbia	1.5	0.1	17.3	33
Slovakia	2.4	0	16.9	30
Slovenia	7	3.4	37.5	42
Spain	6.9	10.6	35.5	67
Sweden	16.7	1.0	27	63
Switzerland	13.7	4.6	35.8	36
Turkey	1.6	0	25.3	4
Ukraine	3.9	0	31	22
United Kingdom	32.5	8.5	42.6	66

NA—not applicable as EMIS only provides aggregated data for these three territories whereas Rainbow Index does not provide aggregated data.

## Data Availability

The data we used is publicly available from the papers and websites cited.

## References

[1] Vivancos R., Anderson C., Blomquist P., Balasegaram S., Bell A., Bishop L., Brown C.S., Chow Y., Edeghere O., Florence I. (2022). Community transmission of monkeypox in the United Kingdom, April to May 2022. Eurosurveillance.

[2] Kupferschmidt K. (2022). Why the monkeypox outbreak is mostly affecting men who have sex with men. Science.

[3] Public Health England (2022). Investigation into Monkeypox Outbreak in England: Technical Briefing 1.

[4] Duque M.P., Ribeiro S., Martins J.V., Casaca P., Leite P.P., Tavares M., Mansinho K., Duque L.M., Fernandes C., Cordeiro R. (2022). Ongoing monkeypox virus outbreak, Portugal, 29 April to 23 May 2022. Eurosurveillance.

[5] Beer E.M., Rao V.B. (2019). A systematic review of the epidemiology of human monkeypox outbreaks and implications for outbreak strategy. PLoS Negl. Trop. Dis..

[6] The EMIS Network (2019). The European Men-Who-Have-Sex-with-Men Internet Survey. Key Findings from 50 Countries.

[7] Pachankis J.E., Hatzenbuehler M.L., Mirandola M., Weatherburn P., Berg R.C., Marcus U., Schmidt A.J. (2017). The geography of sexual orientation: Structural stigma and sexual attraction, behavior, and identity among men who have sex with men across 38 European countries. Arch. Sex. Behav..

[8] Pachankis J.E., Hatzenbuehler M.L., Hickson F., Weatherburn P., Berg R.C., Marcus U., Schmidt A.J. (2015). Hidden from health: Structural stigma, sexual orientation concealment, and HIV across 38 countries in the European MSM Internet Survey. AIDS.

[9] Stojanovski K., King E.J., Amico K.R., Eisenberg M.C., Geronimus A.T., Baros S., Schmidt A.J. (2022). Stigmatizing Policies Interact with Mental Health and Sexual Behaviours to Structurally Induce HIV Diagnoses Among European Men Who Have Sex with Men. AIDS Behav..

[10] Marcus U., Mirandola M., Schink S.B., Gios L., Schmidt A.J. (2021). Changes in the prevalence of self-reported sexually transmitted bacterial infections from 2010 and 2017 in two large European samples of men having sex with men–is it time to re-evaluate STI-screening as a control strategy?. PLoS ONE.

[11] (2021). International Lesbian G, Bisexual, Trans and Intersex Association. Rainbow Europe 2020. https://ilga-europe.org/report/rainbow-europe-2020/.

[12] Rodriguez-Hart C., Nowak R.G., Musci R., German D., Orazulike I., Kayode B., Liu H., Gureje O., Crowell T.A., Baral S. (2017). Pathways from sexual stigma to incident HIV and sexually transmitted infections among Nigerian men who have sex with men. AIDS.

[13] Campbell C.K., Lippman S.A., Moss N., Lightfoot M. (2018). Strategies to increase HIV testing among MSM: A synthesis of the literature. AIDS Behav..

[14] Storm M., Deuba K., Damas J., Shrestha U., Rawal B., Bhattarai R., Marrone G. (2020). Prevalence of HIV, syphilis, and assessment of the social and structural determinants of sexual risk behaviour and health service utilisation among MSM and transgender women in Terai highway districts of Nepal: Findings based on an integrated biological and behavioural surveillance survey using respondent driven sampling. BMC Infect. Dis..

[15] Bailey H., Turkova A., Thorne C. (2017). Syphilis, hepatitis C and HIV in Eastern Europe. Curr. Opin. Infect. Dis..

[16] Bozicevic I., Voncina L., Zigrovic L., Munz M., Lazarus J. (2009). HIV epidemics among men who have sex with men in central and eastern Europe. Sex. Transm. Infect..

[17] Beyrer C., Baral S.D., Van Griensven F., Goodreau S.M., Chariyalertsak S., Wirtz A.L., Brookmeyer R. (2012). Global epidemiology of HIV infection in men who have sex with men. Lancet.

